# A novel noise handling method to improve clustering of gene expression patterns

**DOI:** 10.1186/1471-2105-12-S7-A3

**Published:** 2011-08-05

**Authors:** Anindya Bhattacharya, Rajat K De

**Affiliations:** 1 Center for Integrative and Translational Genomics, University of Tennessee Health Science Center, Memphis, TN, 38163, USA; 2 Machine Intelligence Unit, Indian Statistical Institute, Kolkata, India

## Background

Cluster analysis of gene expression data is a useful tool for identifying biologically relevant groups of genes that show similar expression patterns under multiple experimental conditions. Performance of clustering algorithms is largely dependent on selected similarity measure. Efficiency in handling outliers is a major contributor to the success of a similarity measure. In gene expression data, there may be pairs of genes that have completely different expression values over a few samples under certain experimental condition(s), although they exhibit similar behavior over the other samples. Depending on the algorithms, these outliers are either placed in single element clusters (hierarchical clustering), are allowed to be in a cluster that is more similar compared to others (partitioning clustering) or they may be completely discarded from grouping (density-based, grid-based and graph-based clustering). In all these cases outliers affect the outcome of a clustering result. Measurement errors or conditional changes during microarray experiments may cause a single sample, if not more, differing in expression level to a great extent compared to the other samples. Expression value of the single or a very few outlier samples may cause a gene to be an outlier. We formulate a new weighted function based method to reduce the effect of outliers on similarity measures. The better the similarity measure is in measuring similarity between genes in the presence of outliers, the better the performance of the clustering algorithm will be in forming biologically relevant groups of genes.

## Results

**Figure 1:**
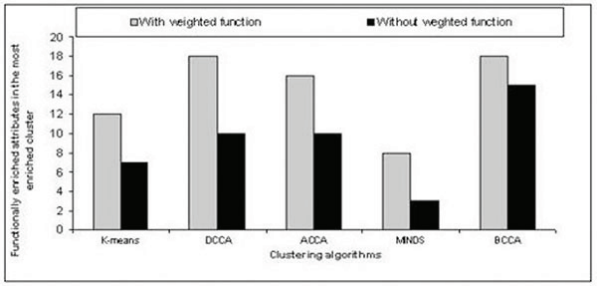
Number of functionally enriched attributes in the most enriched clusters obtained by different clustering and biclustering algorithms.

The effectiveness of the weighted function based method has been demonstrated with the clustering algorithms, *viz.*, K-means [1], Minimization of Disagreement (MIND) [2], Divisive Correlation Clustering Algorithm (DCCA) [3], Average Correlation Clustering Algorithm (ACCA) [4] and Bi-Correlation Clustering Algorithm (BCCA) [5] on a yeast gene expression dataset (Yeast Cheng and Church dataset from Yeast Functional Genomics Database [http://yfgdb.princeton.edu/]). Assessment of the results has been done by using P-values on functional annotations. P-values less than 5.0 × 10^-7^ are reported as enriched functional categories. Figure 1 shows the number of functionally enriched attributes in the most enriched clusters obtained by each of the clustering and biclustering algorithms on the yeast gene expression dataset. The results suggest that the new weighted function based method significantly improves performance of all the cases, in terms of finding biologically relevant groups of genes.
